# Effect of omalizumab on inflammatory markers in COVID-19: an exploratory analysis of the COVID-19 immunologic antiviral therapy with omalizumab (CIAO) trial

**DOI:** 10.3389/fmed.2024.1437322

**Published:** 2024-11-19

**Authors:** Connor Prosty, Michelle Le, Yang Lu, Lauren Khoury, Maxime Cormier, Mathew P. Cheng, Robert Fowler, Srinivas Murthy, Jennifer LY Tsang, Duncan Lejtenyi, Moshe Ben-Shoshan, Elham Rahme, Shirin Golchi, Nandini Dendukuri, Todd C. Lee, Elena Netchiporouk

**Affiliations:** ^1^Faculty of Medicine, McGill University, Montreal, QC, Canada; ^2^Division of Dermatology, Department of Medicine, McGill University, Montreal, QC, Canada; ^3^Department of Epidemiology, Biostatistics, and Occupational Health, McGill University, Montreal, QC, Canada; ^4^Division of Respiratory Medicine, Department of Medicine, McGill University, Montreal, QC, Canada; ^5^Division of Infectious Diseases, Department of Medicine, McGill University, Montreal, QC, Canada; ^6^Department of Critical Care Medicine, Sunnybrook Health Sciences Centre, Toronto, ON, Canada; ^7^Department of Pediatrics, Faculty of Medicine, University of British Columbia, Vancouver, BC, Canada; ^8^Niagara Health Knowledge Institute, Niagara Health, St. Catharines, ON, Canada; ^9^Division of Allergy, Immunology and Dermatology, Department of Pediatrics, Montreal Children’s Hospital, McGill University, Montreal, QC, Canada

**Keywords:** omalizumab, coronavirus, COVID-19, clinical trial, cytokine

## Abstract

**Background:**

The CIAO trial recently demonstrated a probable clinical benefit of omalizumab in the treatment of severe COVID-19; however, the mechanism underlying this benefit remains unclear. Therefore, we sought to longitudinally assess the impact of omalizumab on serum cytokines in CIAO trial patients to determine its mechanism of action.

**Methods:**

Blood samples were collected on days 0, 2, 7, and 14 from patients recruited into the CIAO trial and who consented to this substudy. Blood samples were tested by a panel of 25 inflammatory cytokines, as well as for markers of mast cell activation. Levels of inflammatory biomarkers were compared over time between omalizumab- and placebo-treated patients by generalized linear mixed-effects model. Associations between biomarkers and clinical outcomes were investigated by mixed-effects logistic regression.

**Results:**

Nineteen patients were recruited into this substudy; 10 were assigned to placebo and 9 to omalizumab. Monokine induced by gamma interferon was significantly positively associated with severe COVID-19 (Odds Ratio [OR] = 1.06, 95%CI = 1.00–1.11, *p* = 0.043). Further, omalizumab significantly reduced interleukin-15 (Coefficient = −0.95, *p* = 0.048) and macrophage inflammatory protein-1 (Coefficient = −1.31, *p* = 0.010) levels. However, neither was significant in analyses adjusting for multiple hypothesis testing.

**Conclusion:**

Although limited by a small sample size, these results suggest that omalizumab’s potential benefit in COVID-19 may be mediated independently of modulation of the measured serum biomarkers. Further studies are needed to investigate omalizumab’s mechanism of action in COVID-19.

## Introduction

Coronavirus disease 2019 (COVID-19) has had significant health and economic repercussions globally (1). The pathogenesis of COVID-19 is biphasic involving viral replication and dissemination followed by immune activation (2). Severe COVID-19 is postulated to involve pathological hyperinflammation (2, 3), which has been associated with impaired type I and III interferon (IFN) signaling (4–6). Type II inflammation has also been associated with severe disease (7). A small randomized controlled trial (RCT) studying dupilumab, a monoclonal antibody targeting IL4R-*α*, suggested a possible mortality benefit from inhibition of the type II inflammatory response in severe COVID-19 (8). Therefore, IFN and type II inflammation-targeting agents demonstrate potential as COVID-19 therapies.

Omalizumab is a monoclonal antibody that binds IgE and inhibits its interaction with the FceRI receptor on immune cells (9), used for refractory chronic spontaneous urticaria (10), asthma (11), and nasal polyps (12). Recent data suggest that omalizumab may have antiviral activity (13–15). In asthmatic patients, omalizumab significantly decreased the incidence of upper respiratory tract infections and the duration of viral shedding (15, 16). The postulated mechanism of action was inhibition of IgE-FceRI interaction augmenting type I IFN signaling (17, 18). Omalizumab has also been shown to attenuate type II inflammation/mast cell activation in asthmatic patients (13). In light of this evidence, the COVID-19 Immunologic Antiviral therapy with Omalizumab (CIAO) trial was conducted to assess the efficacy of omalizumab in moderate to severe COVID-19 (19). Despite early termination due to waning recruitment, among the 40 patients enrolled, omalizumab was associated with a 93% probability of reduction in death or mechanical ventilation on day 14 (19). The mechanism by which omalizumab conferred this benefit remains unclear. Thus, we sought to investigate omalizumab’s mechanism of action in COVID-19 by analyzing its impact on longitudinal levels of various biomarkers.

## Methods

### Trial design

This substudy focused on the exploratory endpoints of the CIAO trial (19). Ethics approval was obtained from all participating sites and informed consent was acquired for each subject. Briefly, the CIAO trial was a multi-center randomized double-blind trial comparing a single dose of omalizumab 375 mg plus standard of care vs. placebo with standard of care in patients hospitalized for COVID-19 respiratory illness. The primary outcome was a composite of mechanical ventilation or all-cause mortality at 14 days with secondary endpoints including the time to clinical improvement of COVID-19 evaluated using the WHO 9-point ordinal scale (20). Data for this nested study were obtained from the subset of patients recruited at the McGill University Health Centre (MUHC), who consented to have blood samples drawn during their hospitalization on days 0, 2, 7, and 14 (±2 days).

### Sample processing

Samples were centrifuged and the serum was isolated and stored at −80°C for subsequent batched analysis. The total IgE (IgE) (day 0), tryptase (day 0), and C-reactive protein (CRP) (days 0, 2, 7, 14) assays were performed by the MUHC central laboratory. CD63 expression, a marker of basophil activation, was measured using the CD63 flocast flow cytometry assay (Buhlmann; Schonenbuch, Switzerland), as previously described, on days 0, 2, 7, and 14 (21). A panel of 25 different cytokines was measured for all samples (days 0, 2, 7, and 14) using the cytokine 25-plex human panel immunoassay according to manufacturer’s instructions (Invitrogen; Vienna, Austria).

### Statistical analyses

All statistical analyses were performed using R (4.3.2, Foundation for Statistical Computing, Vienna, Austria). Demographic and clinical data were summarized with descriptive statistics. Mixed-effects logistic regression was used to determine whether measured biomarkers were associated with severe COVID-19 (WHO ordinal COVID-19 score 5–7). In this model, patient identification and study day were random-effect and fixed-effect variables, respectively. Generalized linear mixed-effects models (GLMM) with a gamma distribution were used to model biomarkers over time using the *lme4* package (22). Fixed-effect terms were used for study day and omalizumab assignment and a random-effect term for patient identification. A likelihood ratio test was performed to determine omalizumab’s effect on biomarkers over time. *p*-values in each model were adjusted for multiple hypothesis testing separately using the Benjamini-Hochberg method (Q-value). Line graphs of the cytokine levels over time were plotted using the *ggplot2* package (23).

## Results

### Patient demographics

Of the 40 patients recruited into the CIAO trial, 19 were recruited at the MUHC and consented to blood tests for this substudy. Nine patients were assigned to omalizumab and 10 to placebo. The patient demographics, comorbidities, vaccination status, receipt of concomitant interventions, and WHO COVID-19 ordinal scores are presented in Supplementary Table 1. The median age of the study population was 65.0 years (interquartile range 59.5–80.0) and 31.6% were female. Cardiovascular disease (63.2%), diabetes (52.6%), chronic kidney disease (26.3%), and cancer (21.1%) were the most common comorbidities. The majority (72.2%) of patients were vaccinated against COVID-19. At enrollment WHO ordinal severity scores for COVID-19 were most commonly 4 (36.8%), 3 (31.6%), 5 (21.1%), and 2 (10.5%). Concomitant COVID-19 therapies were received by all patients, including dexamethasone (100.0%), remdesivir (57.9%), tocilizumab (5.3%), and baricitinib (5.3%). The primary outcome, death or mechanical ventilation at day 14, occurred in 4 patients (21.1%).

### Biomarker comparisons

Blood samples were obtained for 100% of participants on day 0, 73.7% on day 2, 52.6% on day 7, and 31.6% on day 14.

In unadjusted analyses, monokine induced by gamma interferon (MIG; OR = 1.06, 95%CI = 1.00–1.11, *p* = 0.043, Q = 0.77) was significantly positively associated with severe COVID-19, but was not significant when adjusting for multiple hypothesis testing (Table 1). None of the remaining biomarkers were statistically significantly associated with the outcome.

**Table 1:** Mixed-effects logistic regression of biomarkers associated with severe COVID-19 (WHO ordinal COVID-19 score 5–7).

Biomarker	OR (95%CI)	*p*-value	Q-value
CD63+ (%)	1.59 (0.91–2.78)	0.10	0.77
CRP (mg/L)	1.03 (0.97–1.10)	0.28	0.95
Eotaxin (pg/ml)	0.72 (0.33–1.57)	0.40	0.95
GM-CSF (pg/ml)	NA	NA	NA
IFN-alpha (pg/ml)	0.98 (0.86–1.13)	0.82	0.95
IFN-gamma (pg/ml)	NA	NA	NA
IL-1 beta (pg/ml)	NA	NA	NA
IL-10 (pg/ml)	NA	NA	NA
IL-12/IL-23 (pg/ml)	1.05 (1.00–1.09)	0.072	0.77
IL-13 (pg/ml)	1.17 (0.70–1.93)	0.55	0.95
IL-15 (pg/ml)	0.97 (0.88–1.08)	0.58	0.95
IL-17A (pg/ml)	0.80 (0.49–1.31)	0.37	0.95
IL-1RA (pg/ml)	1.00 (0.99–1.00)	0.83	0.95
IL-2 (pg/ml)	0.99 (0.93–1.06)	0.82	0.95
IL-2R (pg/ml)	1.00 (1.00–1.00)	0.81	0.95
IL-4 (pg/ml)	1.00 (0.97–1.03)	0.94	0.95
IL-5 (pg/ml)	NA	NA	NA
IL-6 (pg/ml)	0.96 (0.90–1.03)	0.28	0.95
IL-7 (pg/ml)	0.89 (0.71–1.11)	0.29	0.95
IL-8 (pg/ml)	1.00 (0.98–1.02)	0.79	0.95
IP-10 (pg/ml)	1.00 (0.86–1.12)	0.95	0.95
MCP-1 (pg/ml)	1.00 (0.99–1.01)	0.54	0.95
MIG (pg/ml)	1.06 (1.00–1.11)	0.043	0.77
MIP-1 alpha (pg/ml)	1.00 (0.94–1.06)	0.87	0.95
MIP-1 beta (pg/ml)	1.00 (0.98–1.02)	0.72	0.95
RANTES (pg/ml)	1.00 (1.00–1.00)	0.93	0.95
TNF-alpha (pg/ml)	NA	NA	NA
Tryptase (ug/mL)*	0.90 (0.67–1.20)	0.47	0.95
IgE (ug/L)*	1.00 (1.00–1.01)	0.14	0.81

*IgE and tryptase were only performed on day 0; therefore, only a conventional logistic regression was performed for these laboratories.

NA: Not applicable. Too few values above the threshold of detection to perform a logistic regression.

**Figure 1:**
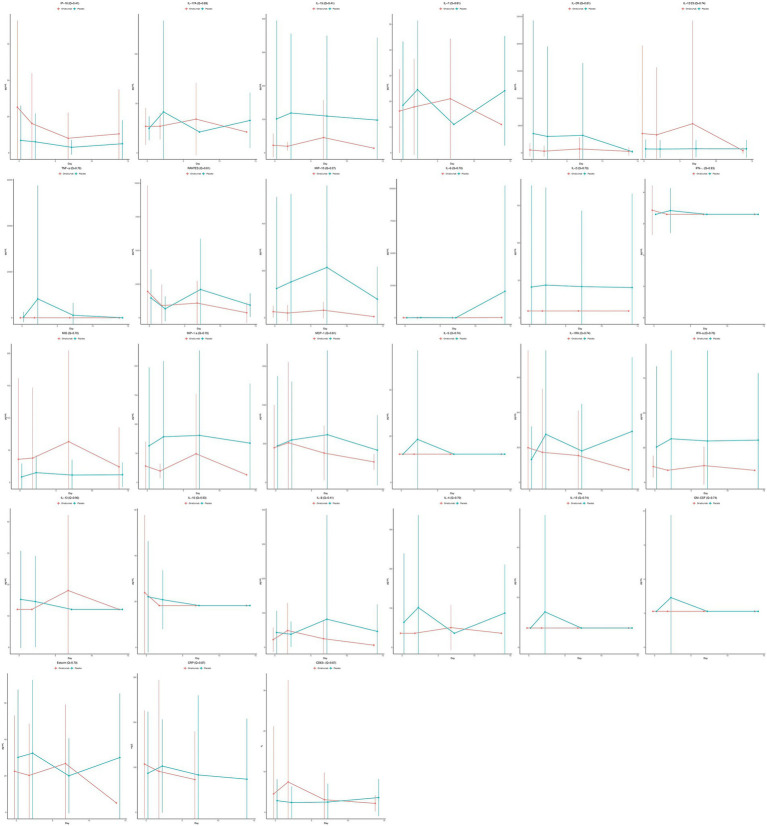
Mean cytokine values over time stratified by treatment assignment, with omalizumab in red and placebo in blue. Solid dots represent the mean and the error bars represent the standard deviations.

Mean biomarker values over time are plotted in Figure 1 for omalizumab vs. placebo. In unadjusted analyses, omalizumab significantly decreased interleukin (IL)-15 (Coefficient = −0.95, *p* = 0.048, Q = 0.41) and macrophage inflammatory protein-1 (MIP-1; Coefficient = −1.31, *p* = 0.010, Q = 0.27); however, these were not statistically significant after adjustment. Receipt of omalizumab did not significantly affect any of the other 25 biomarkers Supplementary Table 2.

## Discussion

After adjustment, we did not find a statistically significant association between the biomarkers studied and disease severity or any significant differences in biomarkers over time as a function of omalizumab treatment.

Previous studies have noted that SARS-CoV2-specific IgE are correlated with COVID-19 severity (24, 25). While these results, combined with omalizumab’s possible efficacy in COVID-19 may suggest a specific role for IgE or type II immunity in the pathogenesis of COVID-19, they may also merely be indicative of global immune dysregulation (7). Indeed, we did not identify any significant associations between disease severity and type II inflammatory cytokines, IFN-*α*, and markers of mast cell activation, in this study with limited sample size. Other studies on the subject revealed conflicting results with one study suggesting a positive association between type II inflammation and COVID-19 mortality (26), whereas another failed to detect a significant association between type II/type I inflammatory cytokine imbalance and death (27).

Our findings suggest that omalizumab’s possible clinical benefit in COVID-19 is mediated by mechanisms other than modulation of serum levels of IFN-*α* or type II inflammation. These results contrast those for dupilumab in COVID-19, which demonstrated a probable clinical benefit of dupilumab and an associated reduction in type II inflammatory cytokines (e.g., eotaxin-3 and YKL-40) compared to placebo (8). Notwithstanding our study’s limitations, there are several alternative explanations for these findings. All patients received concomitant corticosteroids which are known to broadly suppress inflammatory cytokines, including type II inflammation (28), and this may have obscured any omalizumab-induced differences and biased results toward the null. Alternatively, Djukanović et al. demonstrated that, compared to placebo, omalizumab treatment significantly reduced IL-4+ staining cells and eosinophils in bronchial biopsies in asthmatic patients (13). Therefore, omalizumab’s effect may be mediated by immune modulation at the tissue rather than serum level. Additionally, the assay used for cytokine detection in this study does not analyze the levels of certain type II inflammatory cytokines (e.g., IL-9) and type I interferons (e.g., IFN-*β*), which have been shown in other studies to be modulated by omalizumab (18, 29). Consequently, we cannot exclude the possibility that omalizumab affected unmeasured cytokines.

This study has multiple strengths including being conducted within the context of a randomized double-blind RCT, which minimized confounding by severity. Analyses were adjusted for multiple hypothesis testing to reduce the risk of type I error. Despite these strengths, this study is subject to limitations. First, the study population was small which may increase the risk of type II error. Second, the substudy population was further reduced by a loss to follow-up for blood sample collection on later study days, due to death or discharge. Third, many of the cytokine levels fell beneath the limit of detection of the assay, possibly due to concomitant receipt of corticosteroids.

## Conclusion

This study suggests that the possible mortality benefit demonstrated by omalizumab in the CIAO trial could be mediated independently of modulation of serum IFN-*α* and type II inflammatory cytokines. Further studies are necessary to elucidate the mechanism of action of omalizumab in COVID-19 and other viral illnesses.

## Data Availability

The raw data supporting the conclusions of this article will be made available by the authors, without undue reservation.

## References

[1] WHO. Coronavirus (COVID-19) Dashboard. (2023). Available at: https://covid19.who.int (Accessed August 27)

[2] Lamers MM Haagmans BL. SARS-CoV-2 pathogenesis. Nat Rev Microbiol. (2022) 20:270–84. doi: 10.1038/s41579-022-00713-0, PMID: 35354968

[3] del Valle DM Kim-Schulze S Huang HH Beckmann ND Nirenberg S Wang B. An inflammatory cytokine signature predicts COVID-19 severity and survival. Nat Med. (2020) 26:1636–43. doi: 10.1038/s41591-020-1051-9, PMID: 32839624 PMC7869028

[4] Bastard P Rosen LB Zhang Q Michailidis E Hoffmann HH Zhang Y. Autoantibodies against type I IFNs in patients with life-threatening COVID-19. Science. (2020) 370. doi: 10.1126/science.abd4585, PMID: 32972996 PMC7857397

[5] Zhang Q Bastard P Liu Z le Pen J Moncada-Velez M Chen J. Inborn errors of type I IFN immunity in patients with life-threatening COVID-19. Science. (2020) 370. doi: 10.1126/science.abd4570, PMID: 32972995 PMC7857407

[6] Hadjadj J Yatim N Barnabei L Corneau A Boussier J Smith N. Impaired type I interferon activity and inflammatory responses in severe COVID-19 patients. Science. (2020) 369:718–24. doi: 10.1126/science.abc6027, PMID: 32661059 PMC7402632

[7] Lucas C Wong P Klein J Castro TBR Silva J Sundaram M. Longitudinal analyses reveal immunological misfiring in severe COVID-19. Nature. (2020) 584:463–9. doi: 10.1038/s41586-020-2588-y, PMID: 32717743 PMC7477538

[8] Sasson J Donlan AN Ma JZ Haughey HM Coleman R Nayak U. Safety and efficacy of Dupilumab for the treatment of hospitalized patients with moderate to severe coronavirus disease 2019: a phase 2a trial. Open Forum Infect Dis. (2022) 9. doi: 10.1093/ofid/ofac343, PMID: 35959207 PMC9361171

[9] Easthope S Jarvis B. Omalizumab. Drugs. (2001) 61:253–60. doi: 10.2165/00003495-200161020-00008, PMID: 11270941

[10] Maurer M Rosén K Hsieh HJ Saini S Grattan C Gimenéz-Arnau A. Omalizumab for the treatment of chronic idiopathic or spontaneous Urticaria. N Engl J Med. (2013) 368:924–35. doi: 10.1056/NEJMoa1215372, PMID: 23432142

[11] Busse W Corren J Lanier BQ McAlary M Fowler-Taylor A Cioppa GD. Omalizumab, anti-IgE recombinant humanized monoclonal antibody, for the treatment of severe allergic asthma. J Allergy Clin Immunol. (2001) 108:184–90. doi: 10.1067/mai.2001.117880, PMID: 11496232

[12] Gevaert P Omachi TA Corren J Mullol J Han J Lee SE. Efficacy and safety of omalizumab in nasal polyposis: 2 randomized phase 3 trials. J Allergy Clin Immunol. (2020) 146:595–605. doi: 10.1016/j.jaci.2020.05.032, PMID: 32524991

[13] Djukanović R Wilson SJ Kraft M Jarjour NN Steel M Chung KF. Effects of treatment with anti-immunoglobulin E antibody Omalizumab on airway inflammation in allergic asthma. Am J Respir Crit Care Med. (2004) 170:583–93. doi: 10.1164/rccm.200312-1651OC, PMID: 15172898

[14] Cardet JC Casale TB. New insights into the utility of omalizumab. J Allergy Clin Immunol. (2019) 143:923–926.e1. doi: 10.1016/j.jaci.2019.01.016, PMID: 30690050 PMC6939862

[15] Konstantinou GN Podder I Karapiperis D. Omalizumab prevents respiratory illnesses in non-atopic chronic spontaneous urticaria patients: a prospective, parallel-group, pilot pragmatic trial. Clin Transl Allergy. (2023) 13:e12279. doi: 10.1002/clt2.12279, PMID: 37488725 PMC10339798

[16] Esquivel A Busse WW Calatroni A Togias AG Grindle KG Bochkov YA. Effects of Omalizumab on rhinovirus infections, illnesses, and exacerbations of asthma. Am J Respir Crit Care Med. (2017) 196:985–92. doi: 10.1164/rccm.201701-0120OC, PMID: 28608756 PMC5649984

[17] Gill MA Bajwa G George TA Dong CC Dougherty II Jiang N. Counterregulation between the FcepsilonRI pathway and antiviral responses in human plasmacytoid dendritic cells. J Immunol Baltim Md 1950. (2010) 184:5999–6006. doi: 10.4049/jimmunol.0901194, PMID: 20410486 PMC4820019

[18] Gill MA Liu AH Calatroni A Krouse RZ Shao B Schiltz A. Enhanced plasmacytoid dendritic cell antiviral responses after omalizumab. J Allergy Clin Immunol. (2018) 141:1735–1743.e9. doi: 10.1016/j.jaci.2017.07.035, PMID: 28870461 PMC6013066

[19] Le M Khoury L Lu Y. COVID-19 immunologic antiviral therapy with Omalizumab (CIAO) – a randomized-controlled clinical trial. Open Forum Infect Dis. (2024). 11. doi: 10.1093/ofid/ofae102, PMID: 38560604 PMC10977629

[20] Marshall JC Murthy S Diaz J Adhikari NK Angus DC Arabi YM. A minimal common outcome measure set for COVID-19 clinical research. Lancet Infect Dis. (2020) 20:e192–7. doi: 10.1016/S1473-3099(20)30483-7, PMID: 32539990 PMC7292605

[21] Netchiporouk E Moreau L Rahme E Maurer M Lejtenyi D Ben-Shoshan M. Positive CD63 basophil activation tests are common in children with chronic spontaneous Urticaria and linked to high disease activity. Int Arch Allergy Immunol. (2016) 171:81–8. doi: 10.1159/000451084, PMID: 27846634

[22] Bates D Mächler M Bolker B Walker S. Fitting linear mixed-effects models using lme4. J Stat Softw. (2015) 67:1–48. doi: 10.18637/jss.v067.i01

[23] Wickham H. ggplot2: Elegant Graphics for Data Analysis | SpringerLink. (2009). Available at: https://link.springer.com/book/10.1007/978-3-319-24277-4 (Accessed January 18, 2024).

[24] Plūme J Galvanovskis A Šmite S Romanchikova N Zayakin P Linē A. Early and strong antibody responses to SARS-CoV-2 predict disease severity in COVID-19 patients. J Transl Med. (2022) 20:176. doi: 10.1186/s12967-022-03382-y, PMID: 35428263 PMC9012069

[25] Tan C Zheng X Sun F He J Shi H Chen M. Hypersensitivity may be involved in severe COVID-19. Clin Exp Allergy. (2022) 52:324–33. doi: 10.1111/cea.14023, PMID: 34570395 PMC8652637

[26] Baker JR Mahdi M Nicolau DV Ramakrishnan S Barnes PJ Simpson JL. Early Th2 inflammation in the upper respiratory mucosa as a predictor of severe COVID-19 and modulation by early treatment with inhaled corticosteroids: a mechanistic analysis. Lancet Respir Med. (2022) 10:545–56. doi: 10.1016/S2213-2600(22)00002-9, PMID: 35397798 PMC8989397

[27] Pavel AB Glickman JW Michels JR Kim-Schulze S Miller RL Guttman-Yassky E. Th2/Th1 cytokine imbalance is associated with higher COVID-19 risk mortality. Front Genet. (2021) 12:12. doi: 10.3389/fgene.2021.706902, PMID: 34335703 PMC8324177

[28] Braun CM Huang SK Bashian GG Kagey-Sobotka A Lichtenstein LM Essayan DM. Corticosteroid modulation of human, antigen-specific Th1 and Th2 responses. J Allergy Clin Immunol. (1997) 100:400–7. doi: 10.1016/S0091-6749(97)70255-0, PMID: 9314354

[29] Takaku Y Soma T Nishihara F Nakagome K Kobayashi T Hagiwara K. Omalizumab attenuates airway inflammation and interleukin-5 production by mononuclear cells in patients with severe allergic asthma. Int Arch Allergy Immunol. (2013) 161:107–17. doi: 10.1159/000350852, PMID: 23711861

