# Supplementary material for: Effect of omalizumab on inflammatory markers in COVID-19: an exploratory analysis of the COVID-19 immunologic antiviral therapy with omalizumab (CIAO) trial
Source: Front Med (Lausanne). 2024 Nov 19;11:1437322. doi: 10.3389/fmed.2024.1437322 (PMC11611588; doi:10.3389/fmed.2024.1437322)
Supplement: Supplementary file 1 [file Table_1.docx]

**SUPPLEMENTAL TABLES**

**Supplemental Table 1.** Patient demographics, comorbidities, disease severity, and outcomes.

|  | **Omalizumab**  **N (%)**  **n=9** | **Placebo**  **N (%)**  **n=10** | **Overall**  **N (%)**  **n=19** |
| --- | --- | --- | --- |
| **Demographics** | | | |
| Median Age [IQR] | 71.0 [61.0-82.0] | 62.5 [58.8-78.0] | 65.0 [59.5-80.0] |
| Female Sex | 2 (22.2) | 4 (40.0) | 6 (31.6) |
| Asian | 0 (0.0) | 2 (20.0) | 2 (10.5) |
| Black | 1 (11.1) | 1 (10.0) | 2 (10.5) |
| Indigenous | 0 ( 0.0) | 1 (10.0) | 1 (5.3) |
| White | 7 (77.8) | 5 (50.0) | 12 (63.2) |
| Other | 1 (11.1) | 1 (10.0) | 2 (10.5) |
| **Comorbidities** | | | |
| Cancer | 3 (33.3) | 1 (10.0) | 4 (21.1) |
| Cardiovascular Disease | 5 (55.6) | 7 (70.0) | 12 (63.2) |
| Diabetes | 5 (55.6) | 5 (50.0) | 10 (52.6) |
| Chronic Kidney Disease | 1 (11.1) | 4 (40.0) | 5 (26.3) |
| Chronic Pulmonary Disease | 2 (22.2) | 1 (10.0) | 3 (15.8) |
| Atopy | 0 (0.0) | 1 (10.0) | 1 (5.3) |
| **Vaccination Status** | | | |
| Receipt of ≥2 doses of any COVID-19 vaccine* | 6 (66.7) | 7 (77.8) | 13 (72.2) |
| **Concomitant Treatments** | | | |
| Dexamethasone | 9 (100.0) | 10 (100.0) | 19 (100.0) |
| Remdesivir | 5 (55.6) | 6 (60.0) | 11 (57.9) |
| Tocilizumab | 0 (0.0) | 1 (10.0) | 1 (5.3) |
| Baricitinib | 1 (11.1) | 0 (0.0) | 1 (5.3) |
| **WHO Ordinal Scale for COVID-19 Severity at Day 0** | | | |
| 3 - No Supplemental Oxygen | 3 (33.3) | 3 (30.0) | 6 (31.6) |
| 4 - Supplemental Oxygen by Nasal Prongs or Face Mask | 3 (33.3) | 4 (40.0) | 7 (36.8) |
| 5 - Non-Invasive Ventilation or High-Flow Oxygen | 2 (22.2) | 2 (20.0) | 4 (21.1) |
| 6 - Mechanical Ventilation | 0 (0.0) | 0 (0.0) | 0 (0.0) |
| 7 - Mechanical Ventilation and Organ Support (e.g., vasopressors, dialysis, extracorporeal membrane oxygenation) | 1 (11.1) | 1 (10.0) | 2 (10.5) |
| **Clinical Outcome** | | | |
| Death or Mechanical Ventilation on Day 14 | 2 (22.2) | 2 (20.0) | 4 (21.1) |

*Data on vaccination status was unavailable for one patient

**Supplemental Table 2.** Generalized linear mixed model comparison of biomarkers over time as a function of treatment assignment.

| **Biomarker** | **Omalizumab Coefficient** | **P-Value** | **Q-Value** |
| --- | --- | --- | --- |
| CD63+ (%) | -0.18 | 0.74 | 0.87 |
| CRP (mg/L) | -0.21 | 0.74 | 0.87 |
| Eotaxin (pg/ml) | -0.35 | 0.30 | 0.70 |
| GM-CSF (pg/ml) | -0.10 | 0.48 | 0.74 |
| IFN-alpha (pg/ml) | -0.43 | 0.22 | 0.70 |
| IFN-gamma (pg/ml) | -0.0020 | 0.93 | 0.93 |
| IL-1 beta (pg/ml) | -0.15 | 0.49 | 0.74 |
| IL-10 (pg/ml) | -0.016 | 0.90 | 0.93 |
| IL-12/IL-23 (pg/ml) | 0.54 | 0.36 | 0.74 |
| IL-13 (pg/ml) | -0.028 | 0.83 | 0.90 |
| IL-15 (pg/ml) | -0.95 | **0.048** | 0.41 |
| IL-17A (pg/ml) | -0.063 | 0.79 | 0.89 |
| IL-1RA (pg/ml) | -0.29 | 0.42 | 0.74 |
| IL-2 (pg/ml) | -0.45 | 0.27 | 0.70 |
| IL-2R (pg/ml) | -0.41 | 0.66 | 0.81 |
| IL-4 (pg/ml) | -0.31 | 0.29 | 0.70 |
| IL-5 (pg/ml) | -0.12 | 0.49 | 0.74 |
| IL-6 (pg/ml) | -0.83 | 0.30 | 0.70 |
| IL-7 (pg/ml) | -0.13 | 0.61 | 0.81 |
| IL-8 (pg/ml) | -0.72 | 0.060 | 0.41 |
| IP-10 (pg/ml) | 0.99 | 0.060 | 0.41 |
| MCP-1 (pg/ml) | -0.15 | 0.63 | 0.81 |
| MIG (pg/ml) | 0.54 | 0.31 | 0.70 |
| MIP-1 alpha (pg/ml) | -0.61 | 0.18 | 0.70 |
| MIP-1 beta (pg/ml) | -1.31 | **0.010** | 0.27 |
| RANTES (pg/ml) | -0.18 | 0.61 | 0.81 |
| TNF-alpha (pg/ml) | -1.37 | 0.15 | 0.70 |
